# Identification of a novel anthocyanin synthesis pathway in the fungus *Aspergillus sydowii* H-1

**DOI:** 10.1186/s12864-019-6442-2

**Published:** 2020-01-08

**Authors:** Congfan Bu, Qian Zhang, Jie Zeng, Xiyue Cao, Zhaonan Hao, Dairong Qiao, Yi Cao, Hui Xu

**Affiliations:** Microbiology and Metabolic Engineering Key Laboratory of Sichuan Province, Key Laboratory of Bio-Resource and Eco-Environment of Ministry of Education, College of Life Sciences, Sichuan University, Chengdu, 610065 Sichuan People’s Republic of China

**Keywords:** Anthocyanins, Fungus, *Aspergillus sydowii*, Transcriptome, Metabolome, lncRNAs

## Abstract

**Background:**

Anthocyanins are common substances with many agro-food industrial applications. However, anthocyanins are generally considered to be found only in natural plants. Our previous study isolated and purified the fungus *Aspergillus sydowii* H-1, which can produce purple pigments during fermentation. To understand the characteristics of this strain, a transcriptomic and metabolomic comparative analysis was performed with *A. sydowii* H-1 from the second and eighth days of fermentation, which confer different pigment production.

**Results:**

We found five anthocyanins with remarkably different production in *A. sydowii* H-1 on the eighth day of fermentation compared to the second day of fermentation. LC-MS/MS combined with other characteristics of anthocyanins suggested that the purple pigment contained anthocyanins. A total of 28 transcripts related to the anthocyanin biosynthesis pathway was identified in *A. sydowii* H-1, and almost all of the identified genes displayed high correlations with the metabolome. Among them, the chalcone synthase gene (*CHS*) and cinnamate-4-hydroxylase gene (*C4H*) were only found using the de novo assembly method. Interestingly, the best hits of these two genes belonged to plant species. Finally, we also identified 530 lncRNAs in our datasets, and among them, three lncRNAs targeted the genes related to anthocyanin biosynthesis via cis-regulation, which provided clues for understanding the underlying mechanism of anthocyanin production in fungi.

**Conclusion:**

We first reported that anthocyanin can be produced in fungus, *A. sydowii* H-1. Totally, 31 candidate transcripts were identified involved in anthocyanin biosynthesis, in which *CHS* and *C4H*, known as the key genes in anthocyanin biosynthesis, were only found in strain H1, which indicated that these two genes may contribute to anthocyanins producing in H-1. This discovery expanded our knowledges of the biosynthesis of anthocyanins and provided a direction for the production of anthocyanin.

## Background

Anthocyanins are a class of flavonoids that have many agro-food industrial applications such as natural dyes [1]. More recent studies have shown that anthocyanins have potential preventive and/or therapeutic effects on human health, such as improving cardiovascular function and treating obesity [2, 3]. There are six common anthocyanidins: pelargonidin (Pg), peonidin (Pn), cyanidin (Cy), malvidin (Mv), petunidin (Pt) and delphinidin (Dp). Usually, people believe that the anthocyanins could only be derived from the secondary metabolism of plants.

The biosynthesis of anthocyanins in plants has been widely elucidated and well-understood. First, phenylalanine is converted into 4-coumaryl CoA. The conversion is regulated by phenylalanine lyase (PAL), cinnamate hydroxylase (C4H) and 4-coumaroyl CoA ligase (4CL). Second, dihydroflavonol is derived from 4-coumaryl CoA with the help of chalcone synthase (CHS), chalcone isomerase (CHI) and flavanone-3-hydroxylase (F3H). Then, dihydroflavonol is transformed into anthocyanins with the help of dihydroflavonol reductase (DFR) and leucoanthocyanidin dioxygenase (LDOX). After that, the glycosylation of anthocyanins is regulated by flavonoid glycosyltransferase (UGTs) [4, 5]. Finally, Pg and Mv are synthesized from Cy and Dp, respectively, with the help of O-methyltransferase (OMT). Among the genes involved in anthocyanin synthesis, *C4H* is one of the core genes of the phenylpropanoid pathway and mediates the synthesis of secondary metabolites such as anthocyanins [6] and artemisinin [7]. CHS links the phenylpropanoid pathway and the flavonoid pathway as well as plays an important role in the biosynthesis of anthocyanins [8].

In addition to functional genes, other researchers have reported that some regulatory genes played a pivotal role in controlling the synthesis of anthocyanins. Zhou H et al. found that R2R3-MYB can activate the promoters of proanthocyanin synthesis genes to regulate anthocyanin accumulation in peach flowers [9]. Tirumalai V et al. found that micro RNA (miRNA), miR828 and miR858, repressed anthocyanin accumulation though mediating VvMYB114 in grape [10]. Zheng T et al. found that HAT1 regulated anthocyanin accumulation via posttranslational regulation of the MYB-bHLH-WD40 (MBW) protein complex [11]. The findings suggested that we consider the roles of those regulatory genes, including miRNA, lncRNA play in regulating in the biosynthesis of anthocyanins.

As the understanding of metabolomics continues to deepen, several metabolites that were used to be only produced in plants have been produced in microorganisms. For example, betalain in *Penicillium novae-zelandiae* [12]; lawsone, an orange-red pigment, in *Gibberella moniliformis* [13]*;* and Taxol and related taxanes in *Aspergillus niger* [14]. Regarding to the production of anthocyanins from microorganisms, there is no clear confirmation before even though some of the activities of anthocyanin-related genes such as PAL, C4H and 4CL have been detected during *Alternaria sp.* MG1 fermentation [15].

*Aspergillus sydowii,* first named in 1926 by Charles Thom and Margaret Brooks Church [16]*,* was reported as a pathogen of gorgonian corals [17, 18] and found in different habitats where it survives as a soil decomposing saprotroph [19, 20]. Meanwhile *A. sydowii* has been widely studied for its ability to biodegrade agrochemicals and contaminants [21–23]. Moreover, some novel secondary metabolites, such as antidiabetic and anti-inflammatory sesquiterpenoids [24], sesquiterpene and xanthone [25], 2-hydroxy-6-formyl-vertixanthone, 12-O-acetyl-sydowinin A [26], and indole alkaloids [27], were found and identified in *A. sydowii*. These observations demonstrate the capability and complexity of the fungi strain *A. sydowii* in orchestrating the biosynthetic routes of their secondary metabolites.

As reported previously we have successfully isolated and identified a fungi strain H-1 from humus that cultivated bacterial wilt-affected ginger in Chengdu, China, at 2016 [28]. We proved that H-1 belonged to the fungi strain *Aspergillus sydowii* by morphology and phylogeny methods (ITS accession number: MN263259, beta-tubulin accession number: MH426599.1). During the fermentation, we observed a purple pigment has been produced by *Aspergillus sydowii* H-1. In this study, we analyzed and confirmed that the purple components are anthocyanins, explored the anthocyanin synthesis pathway of *A. sydowii* H-1, and investigated the evolutionary relationship between anthocyanin synthesis pathways in fungi and the corresponding pathways in plants. Finally, we also found three regulatory genes were actively involved in the anthocyanin biosynthesis pathway. Our studies firstly discovered that anthocyanin could be produced in the fungi, which will provide new strategies and perspectives for the production of anthocyanins.

## Methods

### Extraction and purification of the purple pigments from *Aspergillus sydowii* H-1

*Aspergillus sydowii H-1* was cultured on Czapek Dox agar medium. Spore suspensions were prepared from 7-day-old culture slants by adding an adequate amount of sterile distilled water. The spore number was 1.6 × 106 cells/mL, which was inoculated into 200 mL of seed culture (Chest’s medium) at 28 °C 180 rpm/min for 60 h. Then, 10 mL of the above mycelial suspension (5% v/v) was inoculated into 200 mL of fermentation medium. The 1 L fermentation medium was composed of 5 g of glucose, 3 g of peptone, 0.5 g of yeast extract, 1 g of KH_2_PO_4_, and 1 g of NaCl. On the 2th (G2) and 8th (G8) day of fermentation, the broth was collected and purified by DM130 macroporous resin. The elution flow rate was 1.5 mL/min and 70% ethanol at a flow rate of 1 mL/min. Then, both the G2 and G8 fermentation broth treated with DM130 macroporous resin were freeze-dried into powder and stored at 4 °C.

### Identifying the purple pigments and determining the biochemical properties during fermentation

Three biochemical properties (fungal biomass, pigment yield and the content of reducing sugar) were monitored from the first day to the 11th day, and all experiments were repeated three times. The dinitrosalicylic acid (DNS) method was used for the quantitative analysis of reducing sugar [29]. The biomass of H-1 was determined by gravimetric analysis after filtering the cell samples through a pre-weighed nylon filter fabric mesh (74 μm porosity) and dried to constant weight at 60 °C. The purple pigment was extracted from the liquid medium through a water-soluble filter of 0.45 μm pore size (Jing Teng, China) and centrifuged at 12,000 rpm for 10 min. The characteristic absorption peak of the purple fermentation broth was scanned at 400~800 nm with a spectrophotometer (Thermo, U.S.A). The content of the purple pigment (extracellular) was quantified indirectly by simply measuring the optical density (OD) at 520 nm, which was the maximum absorption wavelength for the pigment, using a spectrophotometer. Raw data from a time course of biomass, sugar consumption and crude pigment content are shown in Additional file 1: Table S1.

The chemical group of the purple pigment contained was identified by Fourier transform infrared spectroscopy (FTIR). FTIR spectra determination was acquired using Nexus 6700 (Thermo, USA). The above G8 purple lyophilized powder was thoroughly mixed with KBr and pelletized. The resolution of the obtained spectrum was 0.09 cm^− 1^, and the range was 4000–400 cm^− 1^, as described in C.S. Pappas et al. [30, 31].

### Metabolome analysis of *Aspergillus sydowii* H-1 fermentation broth

The lyophilized powder from G2 and the G8 with three independent biological replicates was prepared for downstream analysis. First, 0.1 g of the G2 and G8 powder was extracted overnight at 4 °C with 1.0 mL of 70% methanol aqueous solution and centrifuged at 10,000 rpm/min for 10 min. Following centrifugation at 10,000 g for 10 min, the extracts were absorbed (CNWBOND Carbon-GCB SPE Cartridge, 250 mg, 3 ml; ANPEL, Shanghai, China) and filtered (SCAA-104, 0.22 μm pore size; ANPEL, Shanghai, China) before LC-MS analysis. A quality control sample was prepared by equally blending all samples. During the assay, a quality control sample was run every 10 injections to monitor the stability of the analytical conditions.

The analytical parameters in the LC-ESI-MS/MS system were as follows: HPLC column, Waters ACQUITY UPLC HSS T3 C18 (1.8 μm, 2.1 mm*100 mm); solvent system, water (0.04% acetic acid): acetonitrile (0.04% acetic acid). A gradient elution was performed as follows: 100:0 V/V at 0 min, 5:95 V/V at 11.0 min, 5:95 V/V at 12.0 min, 95:5 V/V at 12.1 min, 95:5 V/V at 15.0 min; flow rate, 0.40 ml/min; temperature, 40 °C; injection volume, 5 μL.

Metabolites were identified on a 6500 QTRAP system (Applied Biosystems, Foster City, CA, USA) equipped with an electrospray source. The ESI source operation parameters were as follows: ion source, turbo spray; source temperature, 550 °C; ion spray voltage (IS), 5500 V; ion source gas I (GSI), gas II (GSII), and curtain gas (CUR) were set at 55, 60, and 25.0 psi, respectively. Instrument tuning and mass calibration were performed with 10 and 100 μmol/L polypropylene glycol solutions in triple quadrupole (QQQ) and LIT modes, respectively.

Qualitative analysis was performed according to the method reported previously [32]. Orthogonal projections to latent structures-discriminate analysis (OPLS-DA) was performed on the identified metabolites. Variable importance in projection (VIP) ≥ 1, fold change ≥2 and *P*-value ≤0.5 were used as the threshold of significantly different metabolites.

### RNA extraction, cDNA library preparation, and RNA sequencing

RNA was isolated, and cDNA libraries were constructed on the second fermentation day and the eighth fermentation day (three replicates for each time point) according to the Illumina HiSeq X-Ten (Illumina, San Diego, CA) RNA library protocol. Library sequencing was performed on a HiSeq X-Ten (Illumina) platform to obtain 150 bp paired-end reads. The raw sequencing reads were submitted to the National Center for Biotechnology Information (NCBI) (BioProject: PRJNA542911).

### RNA-Seq analysis pipeline

Low quality reads were trimmed by Trimmomatic (version 0.36) [33]. The reads that mapped to the known transfer RNAs (tRNAs) and ribosomal RNAs (rRNAs) were removed by searching the Rfam database via Bowtie2 2.3.2 [34]. Then, the trimmed and rRNA-free reads were mapped to *Aspergillus sydowii* CBS 593.65 [35] with Hisat2 (version 2.1.0) [36], and transcripts were assembled with StringTie (version 1.3.3b) [37] by a reference-guided method with default parameters. In order to discover more sequences that are not present in the reference genome, that is, transcripts unique to *A. sydowii* H1, Trinity version 2.8.4 [38] was used to assemble transcripts by the de novo method with the default parameters. De novo assembled transcripts shorter than 300 bp were discarded, and the longest transcript in each cluster (gene) was selected as the representative of the unigene. By comparing the reference-guided sequences with the de novo-assembled unigenes by blastn, the sequences that aligned to reference-guided sequences were removed. The remaining unigenes appeared as the de novo assembly results. The union of the genes obtained by the two methods was used as the final genes in *A. sydowii* H-1. The gene expression levels were calculated and normalized via the expectation maximization method with RSEM version 1.2.31 [39].

In order to obtain the functional annotation of the de novo-assembled unigenes, the coding sequences (CDSs) and the translated protein sequences of the unigenes were predicted with TransDecoder version r20140704 (http://transdecoder.github.io/, accessed 26 Sept. 2018). Then, proteins were functionally annotated by blastp (Camacho et al., 2009) based on queries of functional databases, including the SwissProt database, NCBI nonredundant database and RefSeq database. Pathway annotation of the Kyoto Encyclopedia of Genes and Genomes (KEGG) terms was performed using KOBAS version 3.0 [40], and protein domains were annotated using IterProScan5 version 2.0 (https://github.com/ebi-pf-team/interproscan) against with the Pfam database. Differentially expressed genes (DEGs) between G2 and G8 were analyzed using the DESeq2 package [41]. *P*-value ≤ 0.05 and the absolute value of fold change ≥2 were set as the threshold for identifying significantly differentially expressed genes.

### LncRNA analysis pipeline

First, the NONCODE database [42] was used to characterize the annotated lncRNAs in *A. sydowii* H-1 from the assembled transcripts, but none of the transcripts matched the known lncRNAs. Then, to identify novel lncRNAs, we followed the steps below to filter novel lncRNAs from the newly assembled transcripts. Multiple-exon transcripts were considered to be expressed if they had a TPM (transcripts per million) greater than 0.5. For single-exon transcripts, more rigorously, the TPM was greater than 2. Those foregone coding genes or transcripts with sizes less than 200 nt were filtered out. Last but not least, lncRNA candidates were identified by CPC2 (version 0.1) [43], CNCI (version 3.0, [44], PLEK (version 1.2) [45] and LGC (version 1.0) [46]. Candidate transcripts predicted to have noncoding potential by two or more programs and did not contain any known structural domains were considered the lncRNAs in *A. sydowii* H-1.

To reveal the potential function of the lncRNAs, their target genes were predicted for both trans- and cis-acting functions. Cis-acting, refers to the action of lncRNAs on neighboring target genes. In this study, coding genes ranging from 100 kb upstream and downstream of lncRNAs were searched for cis-acting target genes. The trans role refers to the influence of lncRNAs on other genes at the expression level. RNAplex [47] and LncTar [48] software were used to predict lncRNA target genes that were trans-acting. Finally, the Pearson correlation coefficient between lncRNAs and their target genes was calculated by R language. High confidence pairs (|cor| ≥ 0.7 and *P*-value≤0.5) seemed to be the most likely interaction between the lncRNA and its target gene.

### Phylogenetic trees with 2-oxoglutarate-dependent oxygenases (2-ODD) families

The protein sequences from the 2-ODD family, including the candidate transcripts and known anthocyanin-related genes, were aligned using MUSCLE version 3.8.31 [49] with the default parameters, and the corresponding CDSs were back-translated from the corresponding protein sequences. The conserved CDSs were extracted with the Gblocks method [50]. The bootstrap consensus of the phylogenetic tree was inferred from 100 replicates. Maximum likelihood trees were compiled with RAxML version 8.2.7 software [51] and edited with iTOL (https://itol.embl.de).

### Real-time quantitative PCR (RT-qPCR) validation

Total RNA was extracted from 100 mg of fungal mycelia using TRIzol reagent (Invitrogen, Carlsbad, CA). Reverse transcription was performed using the PrimeScript™RT reagent Kit with gDNA Eraser (TaKaRa). Nine anthocyanin-related genes were selected for RT-qPCR, and the specific RT-qPCR primers were designed with Primer Premier 5 software (Additional file 5: Table S5). Primers for RT-qPCR were synthesized by the Chengdu Qingke Zi Xi Biotechnology Company (Chengdu, China).

With the relative quantitative method, each quantitative reaction was performed in a reaction mixture with a total volume of 25 μL, including 12.5 μL of 2× SYBR Premix Ex Taq TM II (TaKaRa), 2 μL of diluted cDNA template, 1 μL of each primer (10 μM) and 8.5 μL DNase-free water. The amplification was predenatured at 95 °C for 30 s, denatured at 95 °C for 40 cycles for 5 s, and annealed and extended at 60 °C for 34 s. Three technical replicates were tested for each gene, β-tubulin was used as an internal reference gene, and the 2 − ΔΔCT method was used to calculate the relative expression of the genes. All data displays and statistical analyses were performed using GraphPad Prism 5. *P ≤ 0.05, **P ≤ 0.01, ***P ≤ 0.001, are given in the figure legends.

## Results

### The characterization of fermentation and the preliminary identification of anthocyanins

**Fig. 1:**
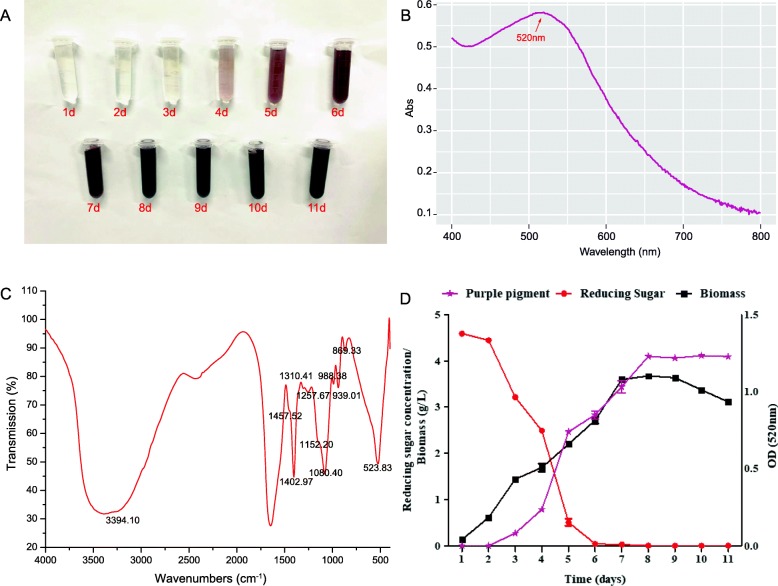
The characteristic of the purple pigment produced by H-1. (**A**) Color change of the *A. sydowii* H-1 fermentation broth from day 1 to day 11. (**B**) Characteristic absorption peak of purple determined with a spectrophotometer. (**C**) Identification functional group of the purple substance by FTIR. (**D**) Time course of biomass, sugar consumption, and crude purple pigment production. Abs, absorbance value; OD, optical density

The fermentation characteristics of *A. sydowii* H-1 were monitored (Fig. 1d). During the day 1 and day 2, *A. sydowii* H-1 was in a growth delay period with slow sugar consumption rate. At this period no pigments were observed. During days 3–7, the fungi were in the logarithmic growth phase. The mycelium grew rapidly with rapid glucose consumption. The UV-visible absorption spectroscopy showed that the characteristic absorption peak at 520 nm gradually increased, indirectly indicating the accumulation of the purple pigments. We observed a maximum pigment absorbance of the fermentation broth at the 8th day of fermentation. The fungi were in a stable phase with slow growth. The weight of the cells decreased in the last three days of culture, and the hyphae began to disintegrate because of autolysis. At the same time, the mycelium no longer produced purple pigments.

According to the growth curve of *A. sydowii* H-1 and the rough production of pigments, the two key time points of *A. sydowii* H-1 fermentation were the 2nd day, the pregrowth period with no significant accumulation of pigments, and the 8th day, the stable period of the cells with the highest accumulation of pigments. Therefore, we selected the fermentation broth and cells on the second (G2) and eighth (G8) days for subsequent metabolome and transcriptome analysis.

With the increasing of fermentation time, the concentration of pigments was gradually increased (Fig. 1a). The absorption spectroscopy of the fermentation broth (Fig. 1b) showed that the fermentation broth had a maximum absorbance at 520 nm, which was consistent with the absorption peak of anthocyanins [52]. We further confirmed the chemical structure by the FTIR spectra of freeze-dried powder derived from the purple fermentation liquid (Fig. 1c). The stretching band at 3394.10 cm^− 1^ corresponds to the OH vibration of the hydroxyl group [53]. The major peaks observed for chitosan were 1647.07 cm^− 1^ (amide I band) [54], and peaks at approximately 800–1150 cm^− 1^ are characteristic of polysaccharides assigned to the C–O valence vibrations and C–O–C stretching vibrations of carbohydrates, including fructose, glucose and glucomannan. Peaks between 1133 and 1457 cm^− 1^ correspond to anthocyanins [55].

We have proved that the absorption spectroscopy and the FTIR assays of the purple pigment were consistent with the characteristics of anthocyanins. Although the consistency could only provide a preliminary structure confirmation of the purple pigments [56–58], we were able to apply metabolomics analysis to further validate the composition of the purple pigment produced by *A. sydowii* H-1 (see below).

### Widely targeted flavonoid metabolomics

To further determine the composition of the purple pigment produced by *A. sydowii* H-1, we defined two time-points during the fermentation process. One is G2, the pre-growth period at day 2 and the other is G8, the stable period at day 8. Since anthocyanins is a type of flavonoid, we performed LC-MS/MS analyses to analyze flavonoid metabolite. As a result, we identified 85 flavones including seventeen flavonols, ten isoflavones, eight flavanones, seven anthocyanins, six polyphenols and thirty-eight other flavones (Additional file 2: Table S2). An OPLS-DA model with R^2^Y(cum) = 0.98 and Q^2^Y(cum) = 0.99 (Additional file 6: Figure S1), was constructed and was able to distinguish the G8 samples from the G2 samples. The results of the permutation test of the OPLS-DA model were R^2^Y(cum) = 0.35 and Q^2^Y(cum) = − 1.25 (Additional file 6: Figure S2). The low values of the Q intercept indicated the robustness of our models and thus showed a low risk of overfitting, indicating that the model was reliable.

**Fig. 2:**
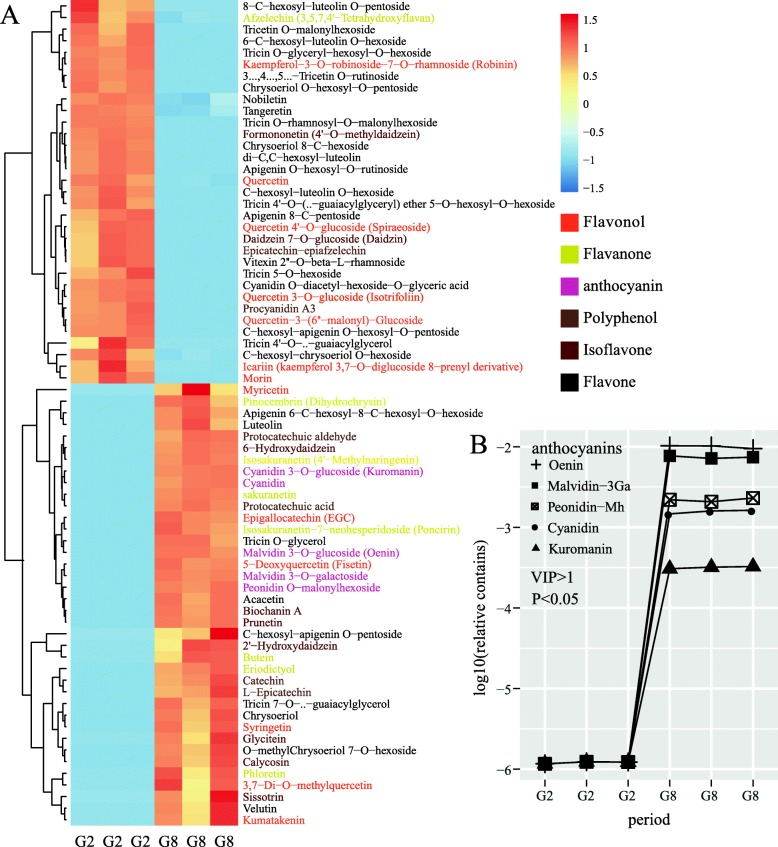
An overview of the changes in flavonoid compounds in *A. sydowii* H-1 in different fermentation stages. (**A**) Differences in the primary metabolite profiles in different fermentation stages; the heatmap color indicates the abundance of each metabolite in different fermentation stages. (**B**) The significant differentially content change in anthocyanin concentration relative to the second day (G2). (G2, the fermentation broth of the second day; G8, the fermentation broth of the eighth day; malvidin-3Ga, malvidin 3-O-galactoside; peonidin-Mh, peonidin O-malonylhexoside)

The metabolites distinguishing the two incubation periods were listed in a heatmap; thirty-nine metabolites were significantly different (VIP ≥ 1, fold change ≥2 and Q-value≤0.05) between G2 and G8 (Fig. 2a) (Additional file 2: Table S2), including five anthocyanins (Fig. 2b): peonidin o-malonylhexoside (peonidin-Mh), cyanidin 3-O-glucoside (kuromanin), cyanidin, malvidin 3-O-galactoside (malvidin-3Ga) and malvidin 3-O-glucoside (oenin). Among those anthocyanins oenin and malvidin-3G were more abundant than the others. Compared the concentration of oenin and malvidin-3G at G8 to G2, there are 8267- and 6147-fold increase, respectively. Because these kinds of anthocyanins have been reported in berries (*Lonicera caerulea*, *Rubus fruticosus, Ribes nigrum* and *Morus alba*), cereals (*Zea mays*) and vegetables (*Brassica oleracea*, *Dioscorea alata, Daucus carota* and *Asparagus officinalis*) [59–61], the LC-MS/MS analysis results have verified that the purple pigment yielded by *A. sydowii* H-1 is anthocyanins.

### Transcriptome sequencing, transcript construction and the analysis of differentially expressed genes

After confirming the composition of the metabolites, RNA-Seq was used to construct the transcripts of *A. sydowii* H-1 in both the second (G2) and eighth (G8) days with three biological replicates. After the removal of adaptor-contaminated, low-quality and rRNA reads, the clean reads from RNA-seq were aligned to *Aspergillus sydowii* CBS 593.65 [35] by Hisat2 version 2.0.4 [36], and the mapped ratio ranged from 78.47 to 91.04%. The mean GC content and Q30 were 53.41 and 94.16%, respectively (Table 1). A high Q30 value indicates that the sequencing data are authentic. Assembly was performed using the reference-guide and de novo method to obtain transcripts as complete as possible (see method). In total, 13,045 gene loci consisting of 15,161 transcripts, including 14,376 reference-guide-derived transcripts and 785 de novo-derived transcripts, were obtained. The transcript levels were estimated with RSEM 1.2.31 [39] software. Pearson correlation analysis between samples performed on the expression matrices of the genes (Additional file 4: Figure S4) showed that there was a difference trend between T5 and other biological repetitions of the G8 period. This may be due to the different growth conditions of fungi in the same fermentation stage. Therefore, the subsequent expression-related analysis will not include T5. Gene differential expression analysis identified 5243 differentially expressed genes (DEGs) (|fold change| ≥2 and *P*-value ≤ 0.05) (Additional file 3: Table S3). To verify the accuracy of RNA-seq, we selected some genes for RT-qPCR. The trend in the expression levels of all selected genes was consistent with the RNA-seq data, which proved that our transcriptome data were authentic. (Fig. 6, Additional file 4: Table S4).

**Table 1:** Transcriptome sequencing data statistics for the RNA sequencing libraries made from different fermentation stages of *Aspergillus sydowii*

Sample	Replicates	GC(%)	Q20(%)	Q30(%)	Reads	Bases	rRNA-free Reads	rRNA-free Bases	rRNA-filtered ratio	Mapped reads	Mapped ratio
G2	T01	53.53	97.87	93.93	22,724,491	6,787,477,124	22,645,402	6,313,271,046	0.38%	20,559,760	90.79%
	T02	53.61	97.96	94.29	21,198,078	6,332,113,218	21,134,990	6,763,845,012	0.32%	19,241,295	91.04%
	T03	53.62	97.77	93.82	25,577,671	7,617,484,750	25,468,697	6,915,217,270	0.46%	22,929,468	90.03%
G8	T04	53.41	98.06	94.52	26,278,445	7,837,542,728	25,480,942	7,585,278,212	3.44%	19,994,895	78.47%
	T05	53.03	98.05	94.28	32,213,122	9,615,419,678	31,182,766	7,599,686,528	3.54%	26,018,900	83.44%
	T06	53.24	97.91	94.13	23,831,027	7,127,497,530	23,121,170	9,307,781,776	3.37%	18,362,833	79.42%

### Identification of anthocyanin-related genes

**Fig. 3:**
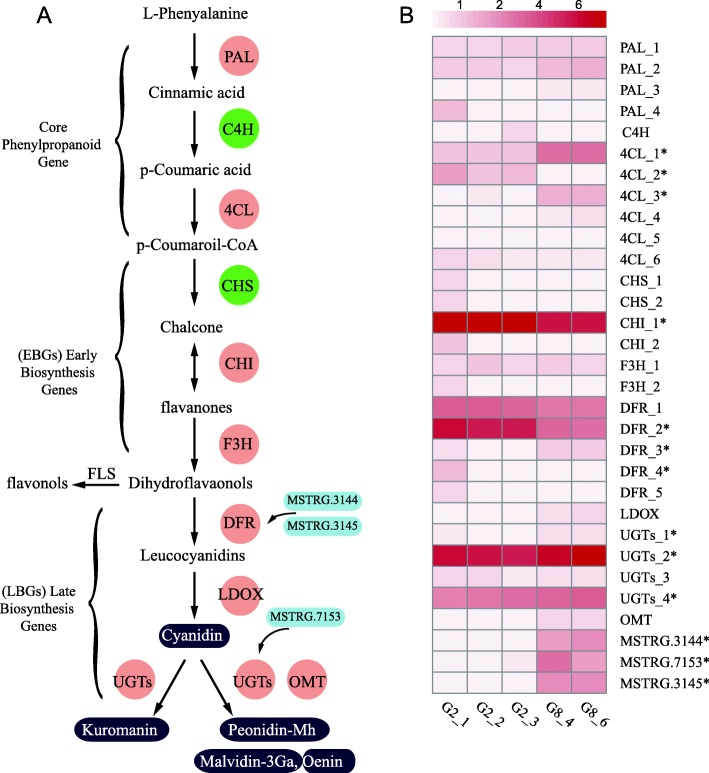
Statistics of gene functions related to anthocyanin metabolism in *A. sydowii* H-1*.* (**A**) Putative anthocyanin synthesis pathway found in *A. sydowii* H-1. Flavonols can be further modified by glycosylation to produce flavonol glycosides. Anthocyanin-related genes can be classified into two major pathways: the phenylpropanoid biosynthesis pathway and the flavonol biosynthesis pathway. The anthocyanin-related compounds identified in *A. sydowii* H-1 are enclosed in purple boxes. *CHS* and *C4H*, which were only found in H-1, are in red circles, and other genes found in the reference genome are in pink circles. Three lncRNAs, which were found in this study, are in light blue boxes (**B**) Heat map of the expression of genes involved in the anthocyanin synthesis pathway in *A. sydowii* H-1. The asterisk indicates a transcript with a significant difference in expression between the two fermentation stages (|fold change| ≥2, *P*-value≤0.05). Malvidin-3Ga, malvidin 3-O-galactoside; peonidin-Mh, peonidin O-malonylhexoside

Although the synthesis pathway of anthocyanins in plants has been studied in details, the anthocyanin-related genes in fungi have not yet been fully explored. In this study, we identified a total of 28 anthocyanin-related genes (Table 2, Fig. 3a), and developed the pathway diagram referring to Guy Polturak et al. [62]. According to Pelletier’s study [63], we divided these genes into early biosynthetic genes (EBGs) and late biosynthetic genes (LBGs). Among these genes, *4CL,* which is the key to the general phenylpropanoid pathway and participates in monolignol biosynthesis through the production of p-coumaroyl-CoA [64]*,* had the largest number of paralogs genes. It is worth noting that *C4H* and *CHS* were only found in our own de novo assembled unigenes. Among them, cinnamate 4-hydroxylase (C4H, EC 1.14.13.11) is the second enzyme of the phenylpropanoid pathway and a member of the cytochrome P450 family. Chalcone synthase (CHS, EC 2.3.1.74) is a key enzyme that catalyzes the first committed step in the flavonoid biosynthetic pathway. Moreover, the best hits of C4H and CHS all blast against plant species genes with very high identity and query coverage (over 95%) (Additional file 5: Table S5). Therefore, similar to the biosynthetic mechanisms in the plant these two genes may contribute importantly to the production of anthocyanins in *A. sydowii* H-1.

**Table 2:** Identification results of anthocyanin-related genes in *A. sydowii* H-1

Pathway	gene id	source	gene symbol	protein name
Phenylpropanoid Biosynthesis related genesPhenylpropanoid Biosynthesis related genes	ASPSYDRAFT_151719	Reference-guided	PAL_1	Phenylalanine ammonia-lyase
	ASPSYDRAFT_57459	Reference-guided	PAL_2	Phenylalanine ammonia-lyase
	ASY002489	denovo	PAL_3	Phenylalanine ammonia-lyase
	ASY009117	denovo	PAL_4	Phenylalanine ammonia-lyase
	ASY000301	denovo	C4H	Trans-cinnamate 4-monooxygenase
	ASPSYDRAFT_28368	Reference-guided	4CL_1	4-coumarate--CoA ligase
	ASPSYDRAFT_86906	Reference-guided	4CL_2	4-coumarate--CoA ligase
	ASPSYDRAFT_89628	Reference-guided	4CL_3	4-coumarate--CoA ligase
	ASPSYDRAFT_94100	Reference-guided	4CL_4	4-coumarate--CoA ligase
	ASPSYDRAFT_188535	Reference-guided	4CL_5	4-coumarate--CoA ligase
	ASY006010	denovo	4CL_6	4-coumarate--CoA ligase
Flavonold Pathway Anthocyanins early biosynthetic genes (EBGs) Flavonold Pathway Anthocyanins early biosynthetic genes (EBGs)	ASY008175	denovo	CHS_1	Chalcone synthase
	ASY009273	denovo	CHS_2	Chalcone synthase
	ASPSYDRAFT_59162	Reference-guided	CHI_1	Chalcone isomerase
	ASY001511	denovo	CHI_2	Chalcone isomerase
	ASPSYDRAFT_203151	Reference-guided	F3H_1	Flavanone 3-dioxygenase/Naringenin
	ASY007110	denovo	F3H_2	Flavanone 3-dioxygenase/Naringenin
Flavonold Pathway Anthocyanins late biosynthetic genes (LBGs)	ASPSYDRAFT_145379	Reference-guided	DFR_1	dihydroflavonol-4-reductase
	ASPSYDRAFT_44997	Reference-guided	DFR_2	dihydroflavonol-4-reductase
	ASPSYDRAFT_72477	Reference-guided	DFR_3	dihydroflavonol-4-reductase
	ASY000353	denovo	DFR_4	dihydroflavonol-4-reductase
	ASY001313	denovo	DFR_5	dihydroflavonol-4-reductase
	ASPSYDRAFT_162316	Reference-guided	LDOX	leucoanthocyanidin dioxygenase/anthocyanidin synthase
	ASPSYDRAFT_91437	Reference-guided	UGTs_1	glycosyltransferase
	ASPSYDRAFT_33013	Reference-guided	UGTs_2	glycosyltransferase
	ASPSYDRAFT_86678	Reference-guided	UGTs_3	glycosyltransferase
	ASY002269	denovo	UGTs_4	glycosyltransferase
	ASY000865	denovo	OMT	O-methytransferase

**Fig. 4:**
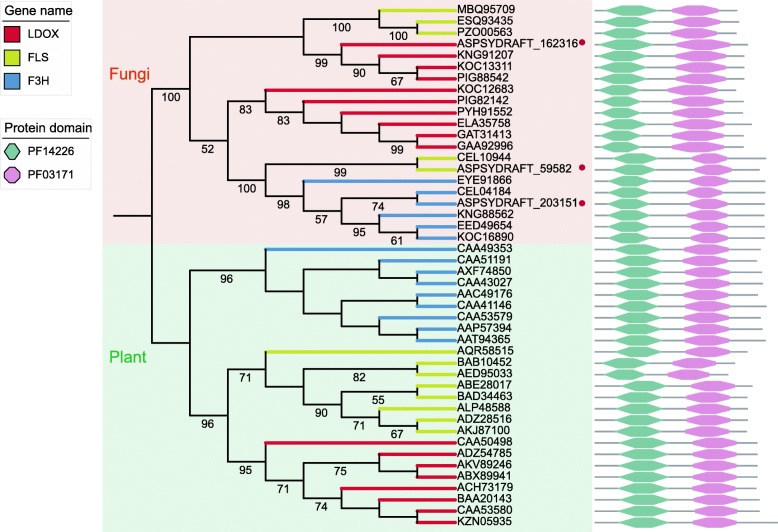
Phylogenetic tree of the 2-oxoglutarate-dependent oxygenases (2-ODD) gene family. The red dots mark the gene in *A. sydowii* H-1. The bamboo green horizontal hexagon and pink octagon represent the relative positions of the common domains of the 2-ODD gene family in this gene

Except for *CHS* and *C4H*, all other genes were found to be the best hits with other fungal species genes. To explore the evolutionary relationship of the remaining anthocyanin-related genes between fungi and plants, the 2-oxoglutarate-dependent oxygenases (2-ODD) family, which includes anthocyanin biosynthesis-related genes, such as leucocyanidin oxygenase gene (*LDOX*), flavanol synthase gene (*FLS*) and flavanone 3-dioxygenase gene (*F3H*), was used for constructing a phylogenetic tree with known homologous sequences in other fungi and plants (Fig. 4). The results showed that all three types of genes were clearly separated according to fungi or plants rather than the type of genes. This separation indicated that the anthocyanin synthesis pathway genes may have evolved separately in plants and fungi. Furthermore, these genes in plants can be clearly divided into different branches according to different gene types but difficult to separate clearly in fungi. This manifestation revealed that these three types of genes may differentiate earlier in plants than in fungi.

### Analysis of the lncRNA genes related to anthocyanins

**Fig. 5:**
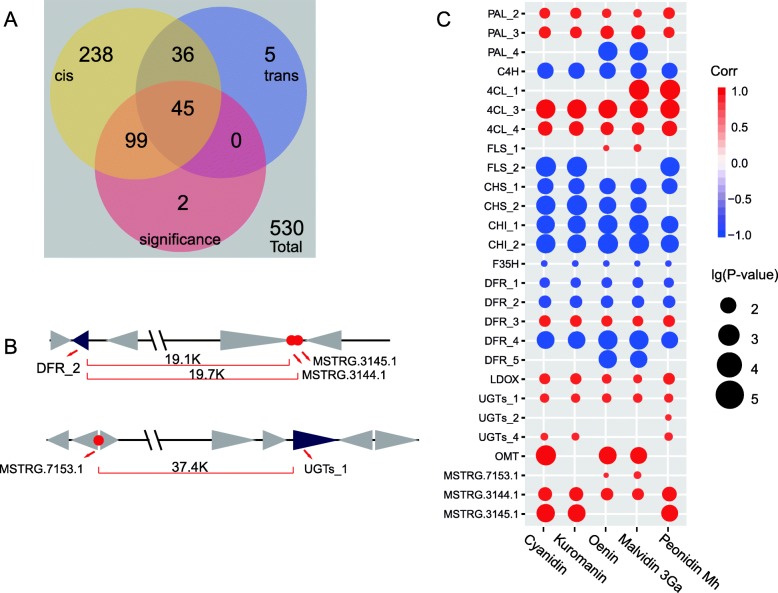
Genes and lncRNAs related to the anthocyanin synthesis pathway. (**A**) Venn diagrams illustrating the number of differentially expressed cis-acting and trans-acting lncRNAs. (**B**) Positional relationship between the lncRNAs (red dots) and the corresponding target genes (purple triangles). The direction of the triangles represents the positive and negative strand in the genome, and the size of the triangle represents the relative size of the locus of the gene. (**C**) Bubble plot showing the correlation between anthocyanin compounds and related structural genes or lncRNAs. Only those with a *P*-value lower than 0.05 are shown. Cis, cis-acting; trans, trans-acting; malvidin-3Ga, malvidin 3-O-galactoside; peonidin-Mh, peonidin O-malonylhexoside

There are several studies have found that lncRNAs performed a variety of functions in different important biological processes [65–67]. However, the role of lncRNAs in regulating anthocyanin synthesis has not been adequately studied. Hence, in this study we examined a total of 530 lncRNAs that were identified in *A. sydowii* H-1. 146 of the lncRNAs showed significantly differential expression between the G2 and G8 samples (|fold change| ≥ 2, *P*-value≤0.05). Among them, 144 lncRNAs regulated target genes by either cis-acting or trans-acting mechanism (Fig. 5a). We followed two steps to filter lncRNAs that may interact with anthocyanin synthesis-related genes: 1) The target genes of the lncRNAs must be an anthocyanin synthesis-related gene (|cor| ≥ 0.7, P-value ≤0.05) and 2) There should be a high correlation (|cor| ≥ 0.7, P-value≤0.05) between the lncRNAs and at least one kind of anthocyanin compound identified by LC-MS/MS. Finally, three lncRNAs appeared to regulate two genes involved in anthocyanin synthesis. One functional gene, ASPSYDRAFT_91437, and three lncRNA genes, MSTRG.7153.1, MSTRG.3144.1, and MSTRG.3145.1, were differentially expressed in G2 and G8 (Fig. 5b, Table 3). The correlation between the function and lncRNA genes involved in anthocyanin synthesis and major flavonoid compounds is presented as a correlogram in Fig. 5c. Interestingly, the highest and most significant positive correlation was found between *4CL* and flavonoids. These results suggest that *4CL* may act as a gatekeeper in the flavonoid biosynthesis pathway and play an important role in regulating the biosynthesis of anthocyanins in *A. sydowii* H-1.

**Table 3:** Identification results of lncRNAs and target genes related to anthocyanin synthesis. The differentially expressed genes (DEGs) are marked with asterisks

gene symbol	id	lncRNA id	Identification of software	acting	position	Correlations	P-value
UGTs_1	ASPSYDRAFT_91437*	MSTRG.7153.1*	LGC/CPC2	Cis-acting	Up-37,447	0.9101	0.0319
DFR_1	ASPSYDRAFT_145379	MSTRG.3144.1*	LGC/CPC2	Cis-acting	Down-19,194	−0.9243	0.0247
DFR_1	ASPSYDRAFT_145379	MSTRG.3145.1*	LGC/CPC2	Cis-acting	Down-19,777	−0.9776	0.004

## Discussion

Anthocyanins are one of the most widespread families of natural pigments in plants and are a class of compounds belonging to flavonoids [68]. Here, we reported a fungus, *Aspergillus sydowii* H-1, could produce anthocyanins under specific fermentation and analyzed the metabolome and transcriptome of these anthocyanins. This is the first time to report the formation of anthocyanins in a fungus as far as we know. This discovery has significant implications for the industrial production of anthocyanins and deepened our understanding of anthocyanins.

The highest content of the six identified anthocyanins was oenin and malvidin-3G, which belong to the malvidin ramification. This result was similar to the anthocyanins components that were reported in blueberries [69], red rice [70] and grapes [71]. Malvidin has been shown to protect against cardiovascular disease [72], and attenuate H_2_O_2_-induced oxidative stress [73] and inflammation [74], demonstrating that this strain has great potential for medical application. Except for malvidin, other content of anthocyanin components in the purple pigments were not exactly the same as that of anthocyanin components reported in above-mentioned plants. This finding suggests that anthocyanin synthesis in fungi is regulated by complex networks different from these in the plants. The specific mechanism in fungi still needs to be further explored.

After trimming, mapping and assembling the transcriptome data, we obtained a total of 15,161 transcripts that included the 28 transcripts involved in anthocyanin synthesis. Interestingly, the expression of LBGs increased after 8 days, while all of EBGs decreased (Fig. 3, 5, 6b, b). The 8th day is the time when anthocyanin accumulated the most in the fermentation broth with declining cumulative rate, and the reduced expression of the relevant genes. These activities increased nutrient consumption and decreased biomass (Fig. 1d). We hypothesized that EBGs in *A. sydowii* H-1 may play a more important role in the yield of anthocyanins than previously imagined.

**Fig. 6:**
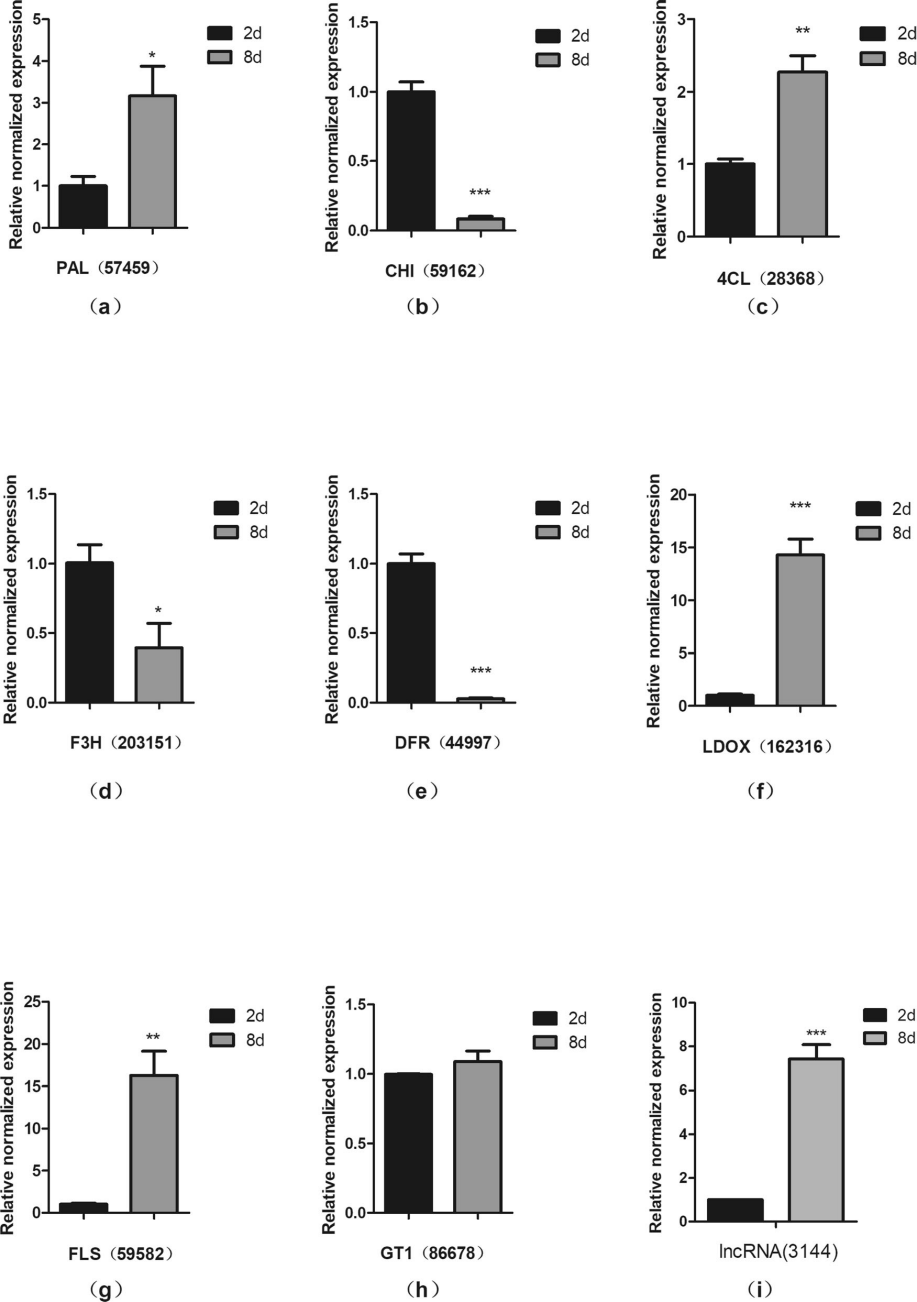
Validation of RNA-seq data by qRT-PCR

*C4H* and *CHS* are vital functional genes in existing studies regarding the process of anthocyanin genes [6, 8, 75]. However, *CHS* and *C4H* could be assembled only by the de novo method, which suggests that the reference genome, CBS 593.65 [35], may not contain these two genes. To rule out errors in gene structural identification, we further mapped the protein sequences of these two genes to the genome using GeneWise [76] with default parameters. However, GeneWise proved the same results, thus negating the possibility that an error occurred in the gene structure annotation. This evidence proves that *CHS* and *C4H* are absent from the reference genome; that is to say, these genes were found in our strain and not in the CBS strain.

Except for the discussion of specificity, we also found these two genes, although only the core domain was assembled, in plant species. The *CHS_1*, *CHS_2* and *C4H* sequences were searched against the NCBI nonredundant database by blastn, which determined that *CHS_2* and *C4H* had the highest identity with *Fagopyrum esculentum*, *Fagopyrum tataricum* and *Acacia mangium* (Additional file 5: Table S5). More interestingly, the alignment method we selected was blastn (nucl-nucl) instead of blastp (prot-prot) or blastx (nucl-prot); such high homology allows us to propose that *A. sydowii* H-1 may have obtained these two genes through plant-fungal horizontal gene transfer (HGT). There are numerous studies suggesting that HGT is an important mechanism in eukaryotic genome evolution, particularly in unicellular organisms [77]. Though phylogenetic inference, Giovanni Emiliani et al. found that the ancestor of land plants acquired a *PAL* via HGT during symbioses with fungi that protect against ultraviolet radiation and capture pigment [78]. Thomas A. Richards et al. defined 14 candidate plant-fungi HGT events by phylogenomic analysis, nine of which showed an infrequent pattern of HGT between plants and fungi, and some kind of genes were involved in transport of the sugar L-fucose and other functions [79]. Meng Li et al. identified 19 fungal genes that had been transferred between fungi and bacteria/plants, which suggests that HGT might have played a role in the evolution and symbiotic adaptation of this arbuscular mycorrhizal fungus [80]. However, due to the incomplete assembly of the transcripts, we will continue to identify the origin of these two genes with the support from complete genomic information.

Among the extensive cortège of plant-associated microorganisms, striking examples of soil microorganisms are mutualistic fungi that have successfully coevolved with their hosts since plants adapted to terrestrial ecosystems [81]. In natural ecosystems, the antagonistic interaction between plants and their pathogens drives a coevolutionary dynamic in which pathogens evolve to avoid plant defense systems and plants evolve to recognize fungal pathogens [82]. Such as Zizhang Wang et al. found a new system, mediating PAMP- and effector-triggered immune responses between peanut and *Aspergillus flavus*, belongs to a three- grade coevolution of plant−pathogen interaction [83]. R. Hung hypothesis that Aspergillus spp. may help plant obtain nutrients beyond its normal capacity and induce systemic changes through extracellular exudates [84]. Asaf Levy et al. found that plant-associated bacteria have ostensibly evolved genes, some of which potentially mimic plant domains or are shared with plant-associated fungi and oomycetes that enable them to adapt to plant environments [85]. Anthocyanins as secondary metabolites protect plant tissues/organs against many abiotic stressors by different mechanisms, such as sunscreen, antioxidant and metal-chelating [86]. Whether the biological meaning of anthocyanins produced in *Aspergillus sydowii* H-1 is a convergent evolution with plants to cope with stress or to mimic the structural domain or function of plant genes to evade plant immune monitoring as mentioned before requires further research.

We further studied regulatory genes, such as long noncoding RNA, which may participate in the anthocyanin synthesis pathway. We predicted that three lncRNAs were involved in the regulation of the anthocyanin-related genes *DFR* and *UGT*. Thus far, it has been reported that these two genes can be regulated in other ways. DFR is a key enzyme that regulates metabolic processes involved in anthocyanin synthesis. It can catalyze dihydroflavonol to produce leucocyanidin. Blue J. Plunkett et al. discovered that the transcription factor MYBA is able to transactivate the DFR promoter in plants without dependence on a coinfiltrated bHLH cofactor [87]. UGT, the final enzyme in anthocyanin biosynthesis, catalyzes glucosyl transfer from UDP-glucose to the 3-hydroxyl group to form stable cyanine glucosides. Which can be upregulated by transcription factors in the preventative effect of Pueraria against cardiac fibrosis [88] or positively regulated by miRNA miR-196a-5p and miR-196b-5p [89]. These studies illustrate the regulatory complexity of *DFR* and *UGT* at the transcriptional level. In this study, we also found three lncRNAs that may regulate *UGTs* and *DFR*: MSTRG.7153.1, may positively regulate *UGTs* and MSTRG.3144.1 and MSTRG.3145.1 negatively regulate *DFR.* These regulators are another possible mechanism for the production of anthocyanins in fungi, providing a possible direction for exploring the mechanism of anthocyanins production.

## Conclusions

We reported that a fungus called *Aspergillus sydowii* H-1 can produce anthocyanins during fermentation. This is the first report that anthocyanins can be produced in non-plant species. Through the study of the synthesis pathway, we found that *CHS* and *C4H* were typical gene existing in the strain and should contribute to fungal anthocyanin production. These findings expanded our understanding of anthocyanin biosynthesis in the nature and in deed provided a new aspect for anthocyanin metabolism.

## Supplementary information

**Additional file 1: Table S1.** Raw data of the time course of biomass, sugar consumption and crude pigment content

**Additional file 2: Table S2.** Metabolome data used in this study. Lists of compounds include ion mode, Rt (min), molecular weight (Da), compounds, class, MEAN value at G2 and G8, VIP, *P*-value, and fold change

**Additional file 3: Table S3.** Differentially expressed genes (DEGs) (P-value < 0.5 and log_2_FC > 1) in G2 and G8. Lists of DEGs include gene ID, length, log_2_FC, *P*-value, count, and annotation (XLS 694 kb)

**Additional file 4: Table S4.** Primers used in this study

**Additional file 5: Table S5.** *CHS* and *C4H* blast results show that the plant-derived gene may be responsible for the production of anthocyanins. Lists of the blast results include gene symbols, query names, subject names, subject species, identity, alignment length, mismatch length, gap openings, query start, query end, subject start, subject end, e-value and score

**Additional file 6: Figure S1.** OPLS-DA score plots generated from OPLS-DA models and different metabolites (DMs) between two time-points. The parameters for the classification were R2Y = 0.98 and Q2Y = 0.99, which were stable and good to fitness and prediction. **Figure S2.** Permutation test was proceeded in order to further validate the model. The R and Q intercept values were 0.35 and − 1.25 after 200 permutations. The low values of Q intercept indicate the robustness of the models, and thus show a low risk of over fitting and reliable. **Figure S3.** The PCA results show that the quality control samples have good repeatability, and the sample mass spectrometry monitoring analysis is stable, and the data repeatability and credibility are high. **Figure S4.** Heatmap of the correlations among samples.

**Additional file 7.** Total transcript fasta sequences obtained in this study

## Data Availability

Supplementary files are included with this submission and contain all the information needed to reproduce the results of this study. The sequencing data generated in current study are deposited in the NCBI SRA database with the BioProject accession: PRJNA542911.

## Abbreviations

2-ODD: 2-Oxoglutarate-dependent oxygenases
4CL: 4-Coumaroyl CoA ligase
C4H: Cinnamate-4-hydroxylase
CHI: Chalcone isomerase
CHS: Chalcone synthase
CPR: Cytochrome p450 reductase
Cy: Cyanidin
DEGs: Differentially expressed genes
DFR: Dihydroflavonol reductase
DNS: Dynitrosalicylic Acid
Dp: Delphinidin
EBGs: Early biosynthetic genes
F3H: Flavanone − 3- hydroxylase
FC: Fold Change
FTIR: Fourier transform infrared Spectroscopy
G2: The fermentation broth of the second day
G8: The fermentation broth of the 8th day
KEGG: Pathway Annotation of the Kyoto Encyclopedia of Genes and Genomes
LBGs: Late biosynthetic genes
LDOX: Leucoanthocyanidin dioxygenase
lncRNA: Long non-coding RNA
Malvidin-3Ga: Malvidin 3-O-galactoside
Mv: Malvidin
NCBI: National Center for Biotechnology Information
NR: Non-redundant protein sequence
OMT: O-methyltransferase
PAL: Phenylalanine lyase
Peonidin-Mh: Peonidin O-malonylhexoside
Pg: Pelargonidin
Pn: Peonidin
Pt: Petunidin
RNAP: RNA polymerase
rRNAs: Ribosomal RNAs
TPM: Transcripts Per Million
tRNAs: Transfer RNAs
UGTs: Flavonoid glycosyltransferase
VIP: Variable Importance in the Projection
