# Supplementary material for: Identification of a novel anthocyanin synthesis pathway in the fungus Aspergillus sydowii H-1
Source: BMC Genomics. 2020 Jan 8;21:29. doi: 10.1186/s12864-019-6442-2 (PMC6950803; doi:10.1186/s12864-019-6442-2)
Supplement: Supplementary file 6 — Additional file 6: Figure S1. OPLS-DA score plots generated from OPLS-DA models and different metabolites (DMs) between two time-points. The parameters for the classification were R2Y = 0.98 and Q2Y = 0.99, which were stable and good to fitness and prediction. Figure S2. Permutation test was proceeded in order to further validate the model. The R and Q intercept values were 0.35 and − 1.25 after 200 permutations. The low values of Q intercept indicate the robustness of the models, and thus show a low risk of over fitting and reliable. Figure S3. The PCA results show that the quality control samples have good repeatability, and the sample mass spectrometry monitoring analysis is stable, and the data repeatability and credibility are high. Figure S4. Heatmap of the correlations among samples. [file 12864_2019_6442_MOESM6_ESM.docx]

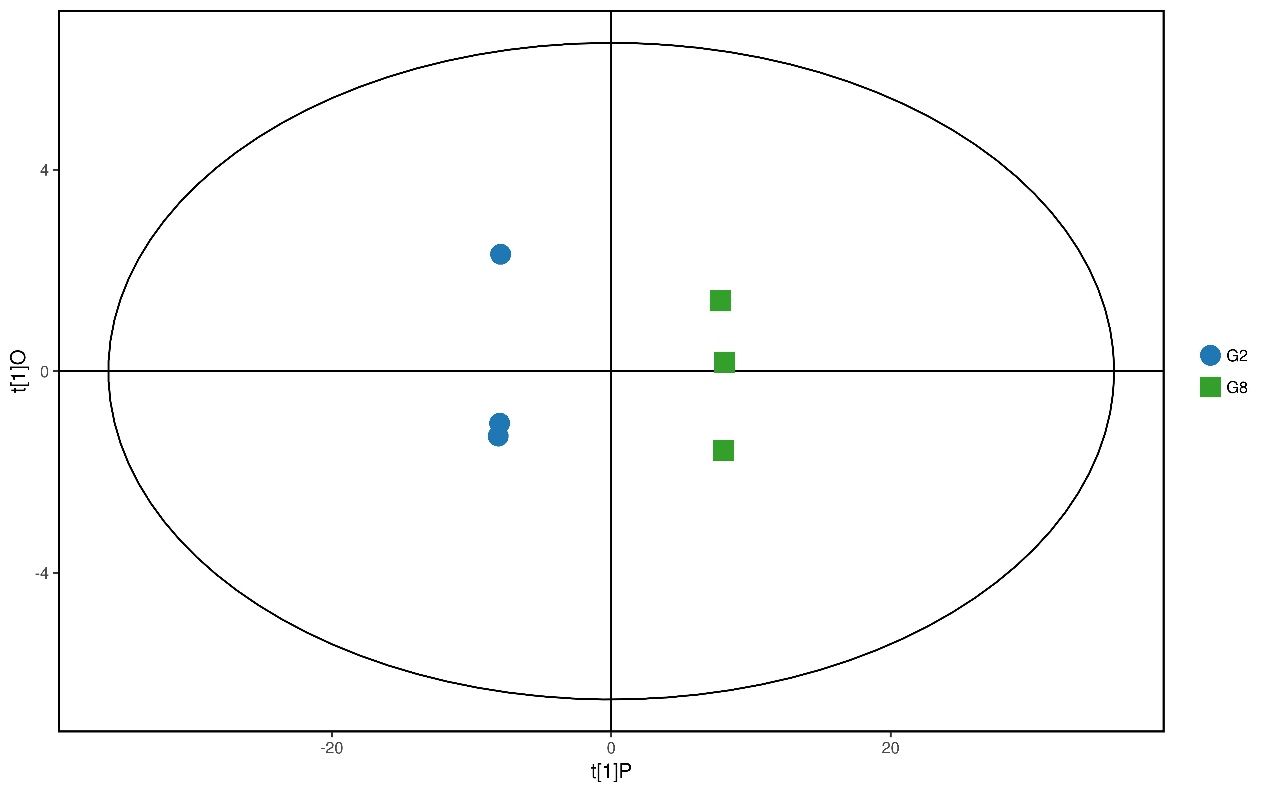


Figure. S1 OPLS-DA score plots generated from OPLS-DA models and different metabolites (DMs) between two time-points. The parameters for the classification were R2Y = 0.98 and Q2Y = 0.99, which were stable and good to fitness and prediction.


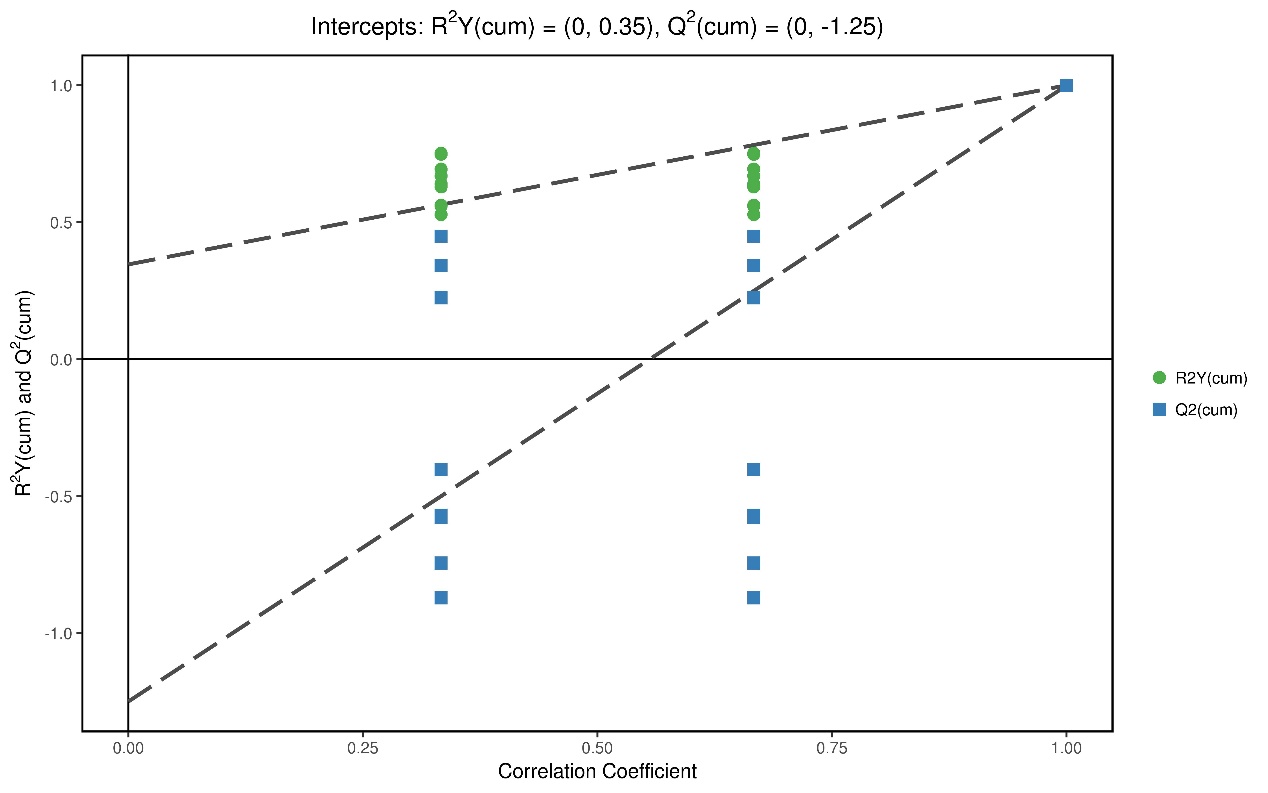


Figure. S2 Permutation test was proceeded in order to further validate the model. The R and Q intercept values were 0.35 and -1.25 after 200 permutations. The low values of Q intercept indicate the robustness of the models, and thus show a low risk of over fitting and reliable.


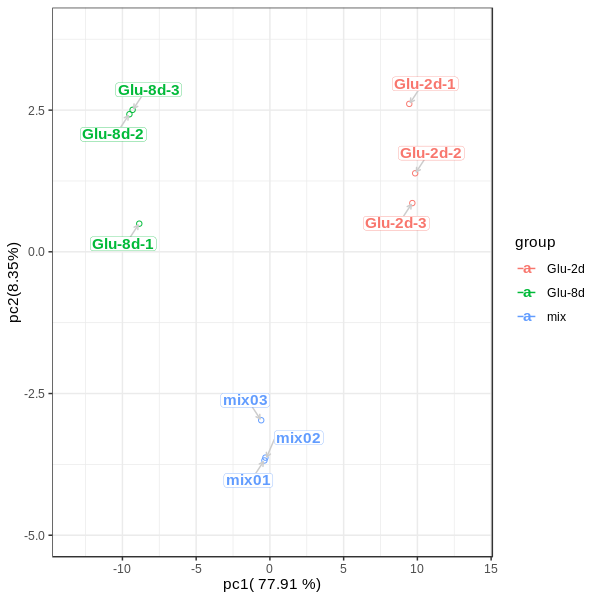


Figure S3. The PCA results show that the quality control samples have good repeatability, and the sample mass spectrometry monitoring analysis is stable, and the data repeatability and credibility are high


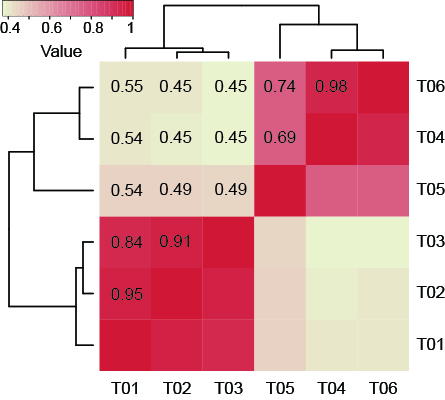


Figure. S4 Heatmap of the correlations among samples
